# Recent Progress in the Applications of Langmuir–Blodgett Film Technology

**DOI:** 10.3390/nano14121039

**Published:** 2024-06-17

**Authors:** Wenhui Gu, Qing Li, Ran Wang, Lexin Zhang, Zhiwei Liu, Tifeng Jiao

**Affiliations:** 1 State Key Laboratory of Metastable Materials Science and Technology, Hebei Key Laboratory of Applied Chemistry, Hebei Key Laboratory of Nanobiotechnology, Hebei Key Laboratory of Heavy Metal Deep-Remediation in Water and Resource Reuse, Yanshan University, Qinhuangdao 066004, China; 2 Hebei Key Laboratory of Safety Monitoring of Mining Equipment, School of Emergency Equipment, North China Institute of Science and Technology, Langfang 065201, China; 3 School of Environmental and Chemical Engineering, Yanshan University, Qinhuangdao 066004, China

**Keywords:** Langmuir–Blodgett film, thin film materials, gas sensors, electrochemistry, biomimetic films, transfer deposition, air–liquid interface

## Abstract

Langmuir–Blodgett (LB) film technology is an advanced technique for the preparation of ordered molecular ultra-thin films at the molecular level, which transfers a single layer of film from the air/water interface to a solid substrate for the controlled assembly of molecules. LB technology has continually evolved over the past century, revealing its potential applications across diverse fields. In this study, the latest research progress of LB film technology is reviewed, with emphasis on its latest applications in gas sensors, electrochemical devices, and bionic films. Additionally, this review evaluates the strengths and weaknesses of LB technology in the application processes and discusses the promising prospects for future application of LB technology.

## 1. Introduction

In recent years, Langmuir–Blodgett (LB) technology has garnered significant attention across various domains due to its development and versatile applications. Originating nearly a century ago, LB film technology enables precise control over molecular arrangement by manipulating surface tension [1,2]. Amphiphilic molecules with aliphatic hydrophobic groups are dissolved in volatile solvents [3]. By meticulously adjusting surface pressure, these molecules form two-dimensionally aligned and ordered monomolecular films at the air/liquid interface, known as Langmuir films (L films) [4,5]. When these films are vertically transferred, they are termed Langmuir–Blodgett films [6].

The foundation of LB technology can be traced back to the pioneering work of Agnes Pockels on surface tension [7]. Irving Langmuir furthered these concepts in 1919 by introducing the Langmuir trough, which facilitated the transfer of organic monolayer lipid molecules from a water surface to solid substrates [8,9]. Subsequent improvements, including the addition of a sliding barrier and the LB trough, enabled the measurement of surface pressure and area (π-A) isothermal curves [10,11]. In 1934, Katharine Blodgett and collaborators extended this technique by depositing Langmuir monolayers onto various solid substrates, enabling the creation of monolayers or multilayers [12,13]. In the 1960s, the potential of LB film technology was further expanded as researchers, like Kuhn, envisioned the construction of complex systems by combining molecules with diverse functions [6,14,15,16]. Since 1983, the International Conference on Ordered Molecular Films (ICOMF) has been held regularly, which promoted the in-depth and rapid development of LB film research [17,18].

LB films possess unique characteristics compared to other films: they can achieve ultra-thin thickness ranging from a fraction of a nanometer to several nanometers, exhibit highly anisotropic lamellar structures, and theoretically offer monomolecular layers with minimal defects [19,20]. This precision at the molecular level makes LB film technology an indispensable tool in molecular engineering. Its unmatched features have fueled the enthusiasm of scientists for LB film research [21,22]. Today, LB film technology remains one of the most effective methods for orderly molecular layer assembly, boasting significant theoretical value and application potential across materials chemistry, optics, electrochemistry, and biomimicry.

In order to make LB membrane technology more widely used in a wider range of fields, this study describes the working principle of LB thin film technology and also evaluates the advantages and disadvantages of LB film technology in applications. The latest research advances in LB thin film technology are summarized, focusing on its latest applications in gas sensors, electrochemical devices, and biomimetic films, and the future application prospects of LB film technology are discussed.

## 2. Principles of Langmuir–Blodgett Film Technology

**Figure 1:**
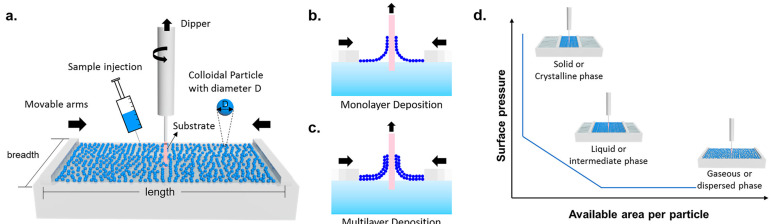
(**a**) Schematic preparation of LB film at the liquid/air interface. Depositing colloidal particles on the substrate to form (**b**) monolayer or (**c**) multilayer LB film. (**d**) Illustration of a typical LB isotherm during compression [23]. Copyright 2022, American Chemical Society.

Langmuir–Blodgett technology is a method for preparing ordered molecular ultra-thin films. Figure 1 shows the schematic process of LB assembly using a vertical pulling method, which relies on interfacial self-assembly [23]. First, the molecules to be deposited are dissolved in a volatile organic solvent, and the solution is added dropwise onto the water surface. After the solvent evaporates, the molecules spontaneously form a monolayer at the air/water interface. By moving a barrier, the molecules can be compressed to the desired density and reach equilibrium. Then, a solid substrate is slowly immersed vertically into the water and then slowly withdrawn, transferring the monolayer uniformly onto the substrate surface. This process can be repeated to build multilayer films. LB technology allows precise control over the arrangement and thickness of each molecular layer, making it suitable for various molecular materials such as lipids, polymers, and nanoparticles. It can achieve highly ordered molecular arrangements, enhancing the functionality and uniformity of the films. Therefore, LB technology is widely used in sensors, electronic devices, and biomaterials.

The Langmuir–Blodgett and Langmuir–Schaefer (LS) techniques are important methods for the preparation of ordered molecular films, and although they both involve the transfer of a molecular layer from a gas–liquid interface to a solid substrate, there are some notable differences in the specifics of their operation and application.

LB and LS technologies each have their own unique advantages and scope of application; LB technology is suitable for applications that require high precision and complex structural control, such as in the field of electronics and sensors. On the contrary, LS technology is more suitable for the preparation of monolayers or for applications that require milder molecular films, such as surface modification and biofilm modeling. Depending on the specific research needs and film properties, choosing the right technology can lead to the best research and application results (Table 1).

This comparison table clearly shows the differences between LB and LS techniques in terms of principles, transfer processes, film structure control, application fields, advantages, and disadvantages, helping to choose the appropriate technique based on specific requirements.

## 3. Application of Langmuir–Blodgett Film Technology in Gas Sensors

Because LB technology has a unique preparation process, a wide range of film-forming materials, and a certain degree of controllable film thickness, it is possible to intentionally design the film structure to serve specific functions and modify it to alter and optimize film properties [24]. Gas sensors represent a major research area within sensor technology. The sensitivity, response speed, and stability of gas sensors are crucial factors for enhancing their performance. Currently, numerous researchers have devoted considerable efforts to the development of modified gas-sensitive materials, mainly focusing on material and film research. LB film technology has emerged as a promising direction of choice in this regard [25]. The integration of LB film technology with gas-sensing materials has made novel advancements in the film-forming process, structural design, and signal detection of thin-film sensors. Gas sensors fabricated using LB film technology have garnered significant attention from scholars due to their superior performance in terms of response and sensitivity feedback.

**Figure 2:**
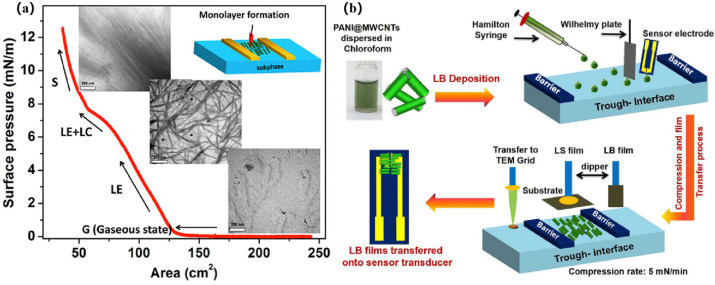
(**a**) The π-A isotherm of PANI@MWCNTs on the air–water interface and corresponding TEM images of different phases. (**b**) LB assembly of PANI@MWCNTs onto the sensor electrodes [26]. Copyright 2020, American Chemical Society.

Sukhananazerin et al. [26] fabricated ultra-thin films of polyaniline-functionalized multi-walled carbon nanotubes (PANI@MWCNTs) with highly ordered structures using the LB technique. Their study revealed that surface functionalization of MWCNTs by PANI reduced the three-dimensional aggregation of CNTs, facilitating the directional assembly of PANI@MWCNTs. Figure 2a depicts the surface pressure-area isotherms of PANI@MWCNTs at the air–water interface. In addition, the formation and durability of the dense single/multilayer structures of PANI@MWCNTs-based LB films were investigated with corresponding TEM images of PANI@MWCNTs at each stage. Remarkably, the attachment of PANI onto the surfaces of MWCNTs was noted to alleviate the three-dimensional clustering of CNTs, fostering the oriented assembly of polyaniline@MWCNTs. PANI@MWCNTs were synthesized via in situ polymerization, as depicted in Figure 2b, illustrating the schematic diagram of PANI@MWCNT LB film formation at the air–water interface and subsequent transfer to the sensor electrode. The highly oriented PANI@MWCNT LB film was initially employed for sensing ammonia gas at room temperature, facilitating directional electron transmission through PANI@MWCNTs. The sufficient molecular accommodation within the directional assembly of the active sensing layer enabled the diffusion of fast analytes, resulting in the high sensitivity of the sensor to NH_3_ gas at room temperature. The extensive orderliness of PANI@MWCNT greatly facilitates the fabrication and development of high-density microelectromechanical system (MEMS) nanoscale sensor arrays for high-performance NH_3_ gas sensors.

**Figure 3:**
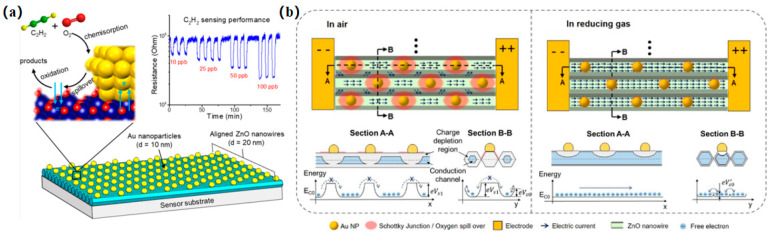
(**a**) Schematic diagram of the LB assembly process of nanostructured thin films, with a thickness of 20 nm, alongside transient response curves corresponding to C_2_H_2_ concentrations ranging from 10 to 1000 ppb. (**b**) Schematic illustration of the electron conduction mechanism of Au@ZnO_LF in air and the reducing gas [27]. Copyright 2020, American Chemical Society.

Complex and dynamic environments present a formidable challenge to the development of effective detection and analysis methods. Consequently, there is a pressing need for the creation of gas detection sensor devices that are convenient, reliable, stable, and sensitive. The progression of stable and exceptionally sensitive thin metal oxide gas-sensitive films is imperative for the development of wireless devices employed in environmental and health monitoring. In a study conducted by Miao et al. [27], ZnO LB films infused with highly dispersed Au nanoparticles were synthesized. The prepared ZnO LB film acts as a very sensitive conductivity switch, showing unmatched sensitivity to acetylene (C_2_H_2_) (see Figure 3). In an ambient air environment, electrons are restricted to a brief path along the nanowires within the conduction channel before encountering a substantial potential barrier (eV_s0_) in the fully depleted area. They are subsequently directed toward neighboring nanowires, overcoming a lesser potential barrier, due to the presence of the high potential barrier. Conversely, in a reducing atmosphere, the depletion depth of charge is diminished as oxygen species are depleted. Consequently, biased electrons can traverse the conduction channel unimpeded, resulting in significantly enhanced conductivity of the sensor. The fabrication of stabilized nanometals on LB films has not only significantly enhanced the sensitivity of sensing films but has also paved the way for numerous applications leveraging metal nanodots, including solar cells, plasmonic resonance waveguides, and biosensors.

**Figure 4:**
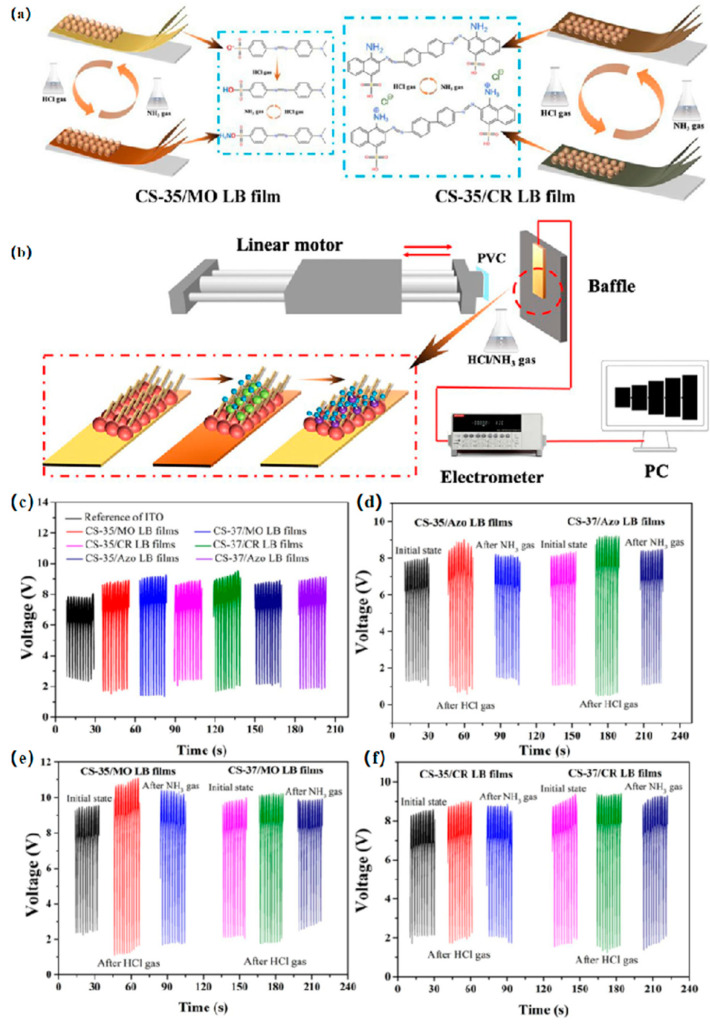
(**a**) Mechanism of LB film response to acid and alkali gases. (**b**) Scheme of the electrical output and sensing capabilities of LB films. (**c**) Voltage output of LB films composed of CS-35/dye and CS-37/dye. Gas sensors equipped with LB films derived from various subphases of (**d**) Azo, (**e**) Methyl Orange (MO), and (**f**) Congo Red (CR) subjected to HCl and NH_3_ gases. Reprinted with permission from [28]. Copyright 2022, American Chemical Society.

Aside from C_2_H_2_, gases, such as ammonia (NH_3_) and hydrogen chloride (HCl), which are widely employed in industrial settings, carry considerable hazards owing to their toxic nature and corrosive properties. These substances pose risks to both human health and environmental integrity. Li et al. [28] proposed a synthesis method for ultra-thin film gas sensors utilizing pH-responsive, self-supplied, and visible composite LB films. As shown in Figure 4a,b, two new carbazole structure sensitizers and several dye molecules were used as sensitizing materials to prepare nano-thick films using LB technology. As depicted in Figure 4c, open-circuit voltages (Voc) were observed at the interface of various carbazole samples/dye LB films. The unaltered indium tin oxide (ITO) surface exhibited a Voc of approximately 6 V, underscoring the function of the prepared LB films as energy gatherers. The output performance of LB films under acidic and alkaline conditions is illustrated in Figure 4d–f. The Voc experienced improvement upon exposure to the CS-35/azobenzene (Azo) composite LB film in an atmosphere containing HCl. When the LB film was exposed to an NH_3_ atmosphere, the Voc recovered. The variation in output performance demonstrates significant gas responsiveness of the prepared LB films, which may be attributed to changes in the surface structure of the LB films. Subsequently, the LB films were assembled into visual sensors capable of pH gas response through simple protonation and deprotonation processes. This novel method presents a fresh direction for the utilization of ultra-thin self-sustaining gas sensors. 

Furthermore, volatile organic compounds (VOCs) pose environmental hazards and threats to human health. LB technology has been employed for the detection of various VOCs. Capan et al. [29] fabricated a multilayer LB film comprising cuproaromatic hydrocarbons and observed enhanced sensitivity and reactivity to VOCs at extremely low concentrations using UV-vis technology. This enabled label-free, real-time, and rapid detection of VOCs. Cuproaromatic multilayer LB films exhibited superior adhesion to chlorinated VOCs compared to aromatic compounds. Erdogan et al. [30] demonstrated that LB sensors based on pyridine-modified cuproaromatics (C_4_P_2_T) exhibited a reduced response to VOCs, including acetone, chloroform, methanol, ethyl acetate, and benzene, suggesting it was sensitive, selective, and suitable for VOC sensing applications.

**Figure 5:**
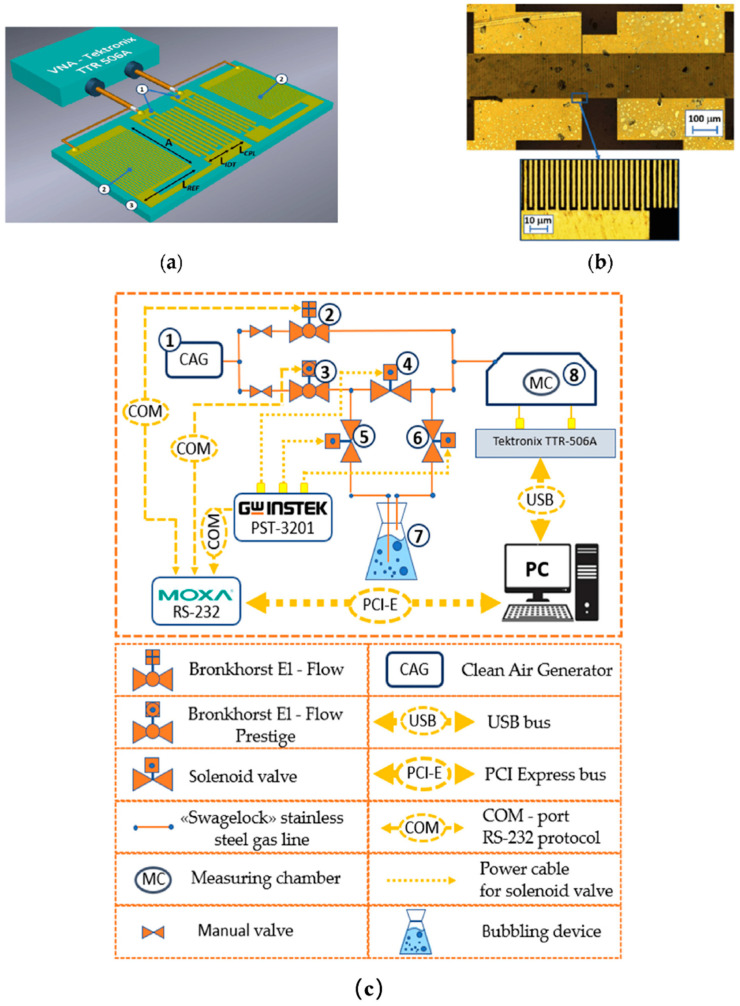
(**a**) Schematic illustration (1: IDT, 2: reflector gratings, 3: piezoelectric substrate) and (**b**) optical image of the central part of the device. (**c**) The scheme of an automated measuring setup (①: air generator; ②, ③: flow meter; ④, ⑤, ⑥: Valve; ⑦: bubbler; ⑧: measuring chamber) [31]. Copyright 2022, MDPI.

Chloroform, commonly used as a solvent in chemical industries and laboratories, poses health risks when inhaled, potentially causing adverse effects on internal organs. Hence, it is essential to develop sensors that can monitor various concentrations of chloroform in the atmosphere. While sensor configurations have been designed for the detection of volatile organic compounds, such as chloroform, achieving selective detection of chloroform is still a challenge. Gorbachev et al. [31] investigated LB films composed of arachidic acid (AA) and stearic acid (SA) as the sensing layer. Figure 5a shows a schematic and Figure 5b shows an optical photograph of the center part of the resonator obtained by optical confocal microscopy with a Lext OLS5000 (Olympus Corp., Tokyo, Japan). When these films are used in two different surface acoustic wave (SAW) devices (a two-port resonator and common delay lines), they can serve as effective coatings for chloroform detection. Notably, with 17 layers of LB film, the amplitude and phase response of the SAW sensors to chloroform surpassed that of toluene and ethanol by several folds. 

Meanwhile, LB technology has found broad application in creating smart biomaterials by incorporating novel peptide molecules. Composite films comprising palmitoyl pentapeptide-4 (PP4) and phthalocyanine dye molecules were synthesized by Li et al. [32]. The TPPS/PP4 LB films demonstrated exceptional responsiveness to acid-alkali gases, offering valuable insights into the fabrication of self-assembled thin films and their utilization in gas sensors.

At the same time, the application of LB technology in the field of metal–organic frameworks (MOFs), which are crystalline structures composed of organic ligands and metal ions with highly tunable and porous structures, has been rapidly developing, and thus has important potential for applications in the fields of gas adsorption, separation, and catalysis. The LB technology can be used to prepare MOF films with specific structures and properties by precisely controlling the self-assembly of the molecular layers and thus optimize their applications in sensors, energy storage devices, and separation technologies. In the future, with further research and application of LB technology in MOFs, it is expected that more versatile and high-efficiency MOF materials will be developed, which will promote their practical application and commercialization in many fields.

These studies demonstrate the significant potential of gas sensors fabricated using LB film technology in enhancing sensor performance and functionality. By managing the fabrication and characteristics of LB films, it is possible to attain heightened sensitivity and specificity toward diverse gases, offering novel remedies for environmental surveillance, industrial manufacturing, and related domains. In summary, the application of LB film technology in gas sensors provides new possibilities for improving sensor performance and functionality. With ongoing technological advancements and refinements, this field is expected to achieve further breakthroughs and advancements.

## 4. Application of Langmuir–Blodgett Film Technology in Electrochemistry

The surface state and electromagnetic properties of electrode materials play pivotal roles in electrochemical reactions. The functionality of electrodes hinges on the precise control of their surface state at the molecular level. Leveraging LB technology facilitates molecular-level assembly design, yielding molecular configurations with directional order and meticulous control over orientation and arrangement. This not only endows electrodes with enhanced functionality but also furnishes the requisite conditions for probing interfacial electron transfer dynamics.

LB technique is instrumental in modifying electrode surfaces. The chemical behavior of the modified electrode closely resembles that of adsorbed molecules. LB-modified electrodes exhibit densely packed films, improving strong interactions among active molecules and exerting a robust shielding effect on the electrode surface [33]. The multifunctionality of LB films makes them a highly promising avenue for advancing electrochemistry. The LB technique offers enhanced precision in self-assembled multilayer structures composed of diverse materials by finely tuning their size and shape. Conductive polymer films prepared using this technique typically exhibit notable conductivity and robust chemical stability.

In a study by Wu et al. [34], homogeneous and meticulously controlled thin films of carboxylated multi-walled carbon nanotubes (c-MWCNTs-Nafion) were assembled and transferred using the LB technique. The resulting microstructures and electrochemical properties surpassed those observed in conventional electrochemical sensors. Microstructural and electrochemical characterization unveiled superior electrocatalytic performance of c-MWCNT-Nafion LB films in electrochemical sensing applications. Subsequently, these thin films were transferred onto the surface of a glassy carbon (GC) electrode to fabricate an electrochemical voltammetric and amperometric sensor for detecting codeine. The thin films prepared in combination with LB technology give the sensor the ability to detect codeine over a specific concentration range and exhibit excellent reproducibility, stability, selectivity, and accuracy.

**Figure 6:**
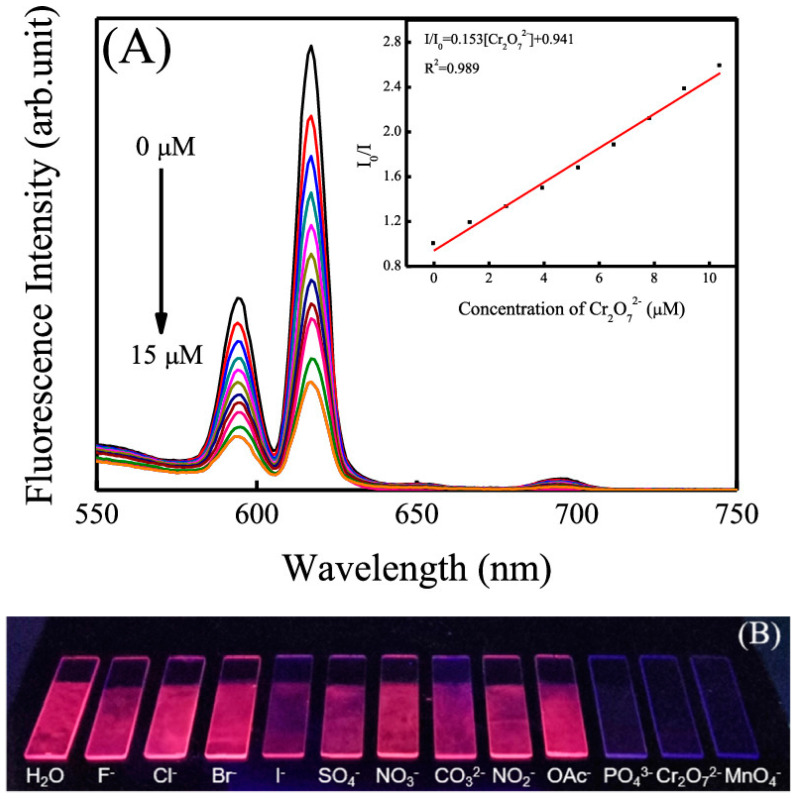
(**A**) Luminescence spectra of nano SiO_2_TPy@EuDPA LB films with different concentrations of Cr_2_O_7_^2−^. (**B**) Photographs of nanoSiO_2_TPy@EuDPA LB films immersed in different anion aqueous solutions under UV light irradiation at 254 nm. The inset shows the Stern–Volmer plot of Cr_2_O_7_ [35]. Copyright 2020, American Chemical Society.

Ma et al. [35] introduce a synthetic approach for fabricating visible luminescent probes at interfaces using Eu^3+^ complex functionalized silica nanocomposites and LB technology. The films obtained by utilizing the LB technique exhibit closely spaced nanocomposite arrays, resulting in improved uniformity and stability, as well as enhanced emission strength and lifetime. Leveraging its exceptional optical properties, these probes serve as straightforward, environmentally friendly, and cost-effective luminescent sensors capable of visually and quantitatively identifying various common toxic anions such as Cr_2_O_7_^2−^, MnO^4−^, and PO_4_^3−^. Furthermore, the effect of ionic concentration on the fluorescence intensity by varying the input of Cr_2_O_7_^2–^ is evaluated and shows a linear relationship in a low concentration region of 0–10 μM (R^2^ = 0.989), as shown in Figure 6, LB film-based materials demonstrate higher Ksv values (1.53 × 10^5^ M^−1^), lower detection limits, and superior recyclability, underscoring their practical utility.

**Figure 7:**
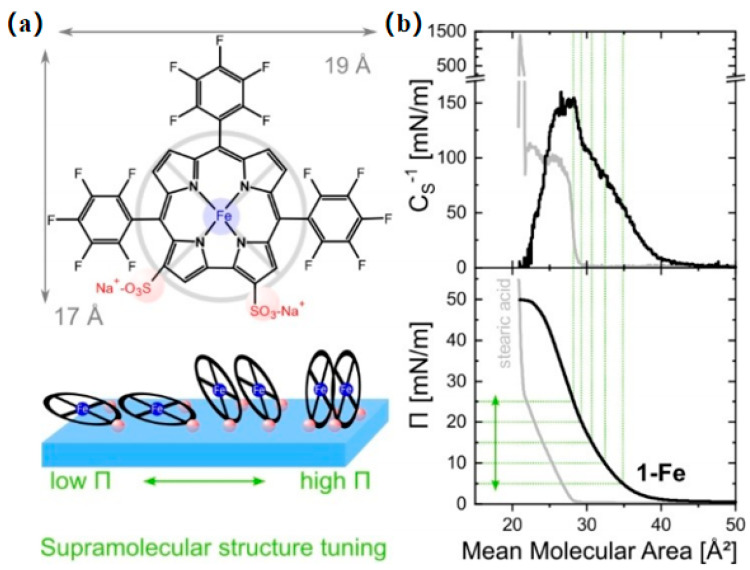
(**a**) Molecular structure of 1-Fe and reduced accessibility of the Fe-binding site with increasing surface pressures (π). (**b**) Surface pressure area isotherms and compression modulus of 1-Fe and reference amphiphilic stearic acid. The dashed lines indicate the average molecular area and surface pressure at deposition, respectively [36]. Copyright 2023, Wiley Online Library.

In a study by Graewe et al. [36], the LB technique was employed to assemble amphiphilic iron(III) molecules into well-defined quasi-two-dimensional molecular monolayers and their stacks at the air–water interface for nitric oxide (NO) detection (Figure 7). The thinner surface functionalization facilitates efficient electronic communication between the layers and the (semi-)conductor. This discovery highlights the advantages of employing the LB technique for assembling sensor molecules into monolayers at the water–air interface.

LB films have emerged as promising materials for the development of capacitors, offering fast response, high sensitivity, immunity to interference, stability, and reproducibility in electrochemical sensors. Leveraging ordered multilayer structures and precise control over morphology and molecular interactions, LB techniques enable the utilization of diverse materials, including carbon nanomaterials, nanoparticles, conducting polymers, and phospholipids [37,38,39,40,41,42]. 

**Figure 8:**
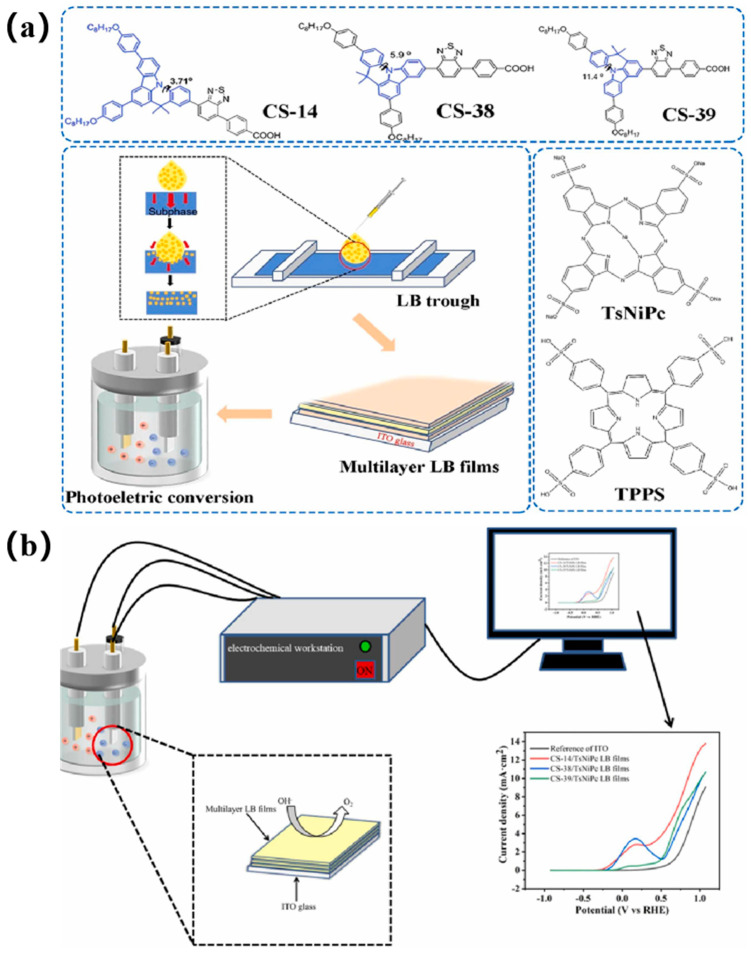
(**a**) Schematic interfacial self-assembly and electrochemical applications of CS/dye composite LB films. (**b**) Schematic diagram of the water electrolysis process of CS/dye molecule multilayer LB composite film. Reprinted with permission from [43]. Copyright 2023, Elsevier.

Bian et al. [43] harnessed the LB technique to fabricate novel carbazole-based organic molecule (CS-14, CS-38, and CS-39) complexes with nickel(II) phthalocyanine-tetrasulfonic acid tetrasodium salt (TsNiPc) and TPPS subphases, resulting in multilayered films. These films were transferred onto different substrates using the horizontal stretching method for subsequent characterizations (Figure 8). The self-assembled LB film electrode demonstrated remarkable photoelectrochemical performance and enhanced electrochemical efficiency, indicating the potential of LB films in developing photoelectrically functional ultra-thin film devices.

Divagar et al. [44] explored the energy storage behavior of N-Vinylpyrrolidon (NVP)–manganese dioxide (MnO_2_) nanocomposite films with varied component ratios, highlighting the superior specific capacity and cycling stability of the 3:1 NVP-MnO_2_ (NM_3_) composition, proving the applicability of NM_3_ in electrochemical energy storage devices.

**Figure 9:**
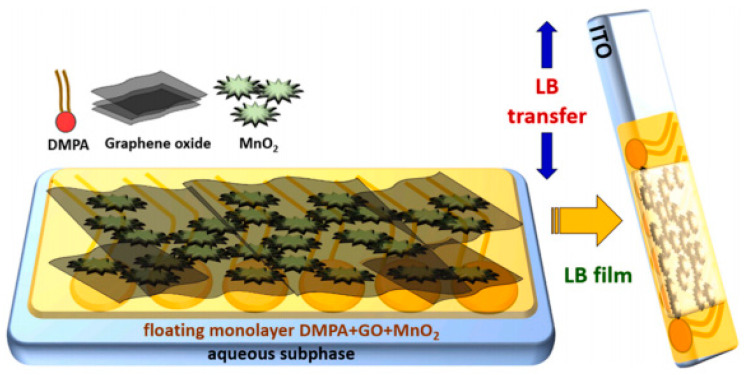
Schematic illustration of the fabrication process for an LB film containing a hybrid nanostructured DMPA + GO/MnO_2_ on an ITO electrode [45]. Copyright 2023, Elsevier.

Scholl et al. [45] synthesized graphene oxide (GO)/MnO_2_ and dimyristoylphosphatidic acid (DMPA) nanocomplexes on conductive electrodes, as illustrated in Figure 9. This pioneering study on LB films of GO/MnO_2_ nanostructures revealed promising performance, positioning them as viable candidates for energy storage applications in supercapacitor bio-devices.

**Figure 10:**
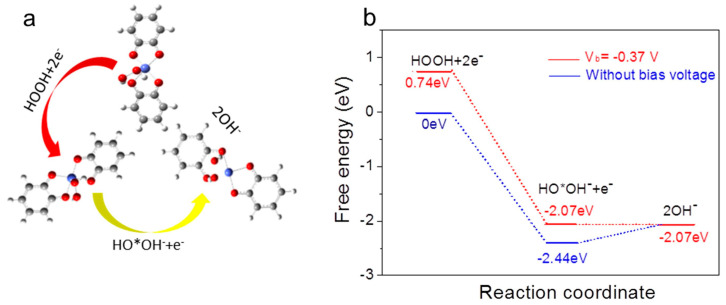
(**a**) The atomic model of [Co_3_(HOB)_2_]n and the proposed H_2_O_2_ reduction pathway. (**b**) Schematic energy profiles for the H_2_O_2_ reduction pathway in alkaline media [47]. Copyright 2021, Elsevier.

The LB technique enables the deposition of nano-monolayers onto substrates of different properties and shapes and, therefore, has certain advantages in terms of structural properties. It has also been widely studied due to its selectable substrate structure and excellent catalytic activity in many reactions. Medina-Plaza et al. [46] developed a nanostructured electrode using the LB technique to prepare two electrocatalytic materials (functionalized gold nanoparticles and lutetium bisphthalocyanine), which were found to be synergistic in improving electrocatalysis for hydroquinone detection. Zhang et al. [47] prepared two-dimensional MOF (metal–organic backbone) [Co_3_(HOB)_2_]_n_ films using the LB method combined with the layer-by-layer growth technique. The atomic model of [Co_3_(HOB)_2_]_n_ and the proposed H_2_O_2_ reduction pathway are shown in Figure 10, along with schematic energy profiles of the H_2_O_2_ reduction pathway in alkaline media. It was found that the conductive metal–organic LB films with fewer layers as electrocatalysts could accelerate H_2_O_2_ decomposition and achieve ultra-low detection limits of H_2_O_2_, with the advantages of fast response, high sensitivity, stability, and reproducibility. Ning et al. [48] investigated floral platinum nano-clusters (FPTNCs) and GO for catalytic synergistic effects. Heterogeneous films consisting of Pt, N-Chlorosuccinimide (NCS), and monolayer GO sheets were prepared by the LB technique, which served as model catalysts for methanol electrooxidation reaction and provided guidance for the synthesis of multiphase catalysts.

The diverse properties of LB technology render it a valuable instrument for advancing next-generation batteries. Battery research typically encompasses various topics spanning multiple scales, including electrode materials [49,50,51], electrode–electrolyte interfacial phases [52,53], and electrolyte systems [54,55,56]. The LB technique has a wide range of applications in battery research. Kim Hyeri et al. [57] prepared graphene nanosheet (GNS)/single-walled carbon nanotube (SWNT) composites using the LB technique and realized that the hybrid structure of graphene and SWNTs was accurately controlled at the liquid–gas interface. The functionalized SWNT Langmuir monolayer anchors monolayer GNSs suspended in water through Coulombic interactions at the interface. This GNS/SWNT hybrid multilayer electrode can be a promising anode material for lithium-ion batteries with a high specific capacity, excellent power capability, and outstanding cycling performance.

In addition, the LB technique can be used to directly fabricate nanomaterial composite electrodes. Ramasamy et al. [58] used the LB technique to prepare tin oxide/reduced graphene oxide (SnO_2_/RGO) nanocomposites as anodes for lithium-ion batteries. The initial discharge capacity of the anode with no binder and additives was 1091 mAh g^−1^, and the Coulombic efficiency was maintained at 62.5% after 20 cycles. The synergistic interaction between SnO_2_ nanoparticles and RGO layers enables the realization of such performance, allowing it to accommodate significant volume changes while maintaining a conductive network. Eom et al. [59] fabricated graphene-like porous two-dimensional Co_3_O_4_ assemblies templated with graphene oxide LB films, where Co_3_O_4_ particles catalyze the degradation of graphene oxide template films, resulting in the production of Co_3_O_4_ nanofilm. The material provided a reversible capacity of 1279.2 mAh g^−1^ over 50 cycles. 

## 5. Application of Langmuir–Blodgett Film Technology in Biomimetic Films

Biologists have long hoped to use LB technology to prepare biomaterials. Single-molecule films have many similarities with biological films that exist in nature. Biological films contain substances such as lipids, proteins, and small amounts of sugars. There are many varieties of lipids, such as cholesterol and phospholipids, which are also amphiphilic compounds that can form a bilayer structure called bilayer Langmuir–Blodgett film (BLB film) [60]. Therefore, this property provides an idea for the artificial preparation of biomimetic films by LB technology, which is used to assemble various organic molecules, such as phospholipids and proteins, to imitate the structure of biological films and to explore the application of biomimetic LB functional films, which will provide further understanding for people with a background in life sciences.

**Figure 11:**
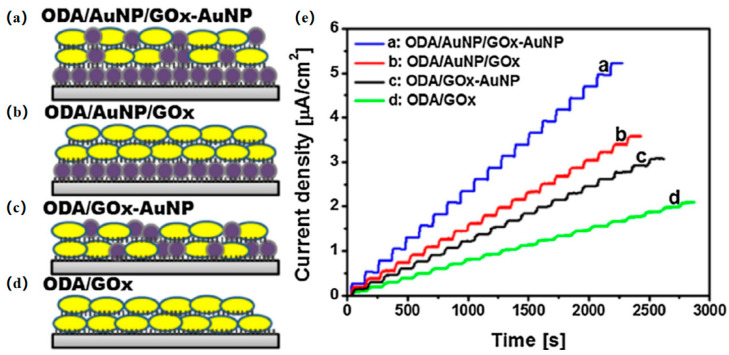
Schematic structures of composite LB films: (**a**) GOx-AuNP, (**b**) AuNP/GOx, (**c**) AuNP/GOx-AuNP, (**d**) GOx. (**e**) Measurement of the electrochemical response of various GOx LB films to continuous glucose injections (0.5 mM increments per injection) [61]. Copyright 2016, Elsevier.

LB films have the potential to be developed as biosensors, and it is thought that the incorporation of enzyme molecules into LB films will result in solid elements with novel functions. Most biomimetic sensors made from LB films contain monolayers or multilayers of lipid organization that serve as suitable substrates to maintain or enhance film activity. Among them, LB nanofilms have been used in enzyme-based biosensors for about a decade, and Wang et al. [61] prepared glucose oxidase/Au nanoparticle composite LB film for glucose-sensing performance (Figure 11). Subsequently, Kuo et al. [62] prepared Ag nanocrystalline LB films for the detection of glucose in surface-assisted laser desorption/ionization mass spectrometry (SALDI MS), which was found to have the highest signal intensity, high sensitivity, and good reproducibility. Gorbachev et al. [63] used the LB technique to prepare an immobilized glucose oxidase (GOx) with 1,2-dipalmitoyl-stannous-glycerol-3-phosphoethanolamine (DPPE) monolayer film and found that the immobilization of GOx enzyme molecules on LB DPPE films increased the conductivity of LB films with increasing glucose concentration, making it possible to develop glucose sensors for higher concentrations. With the use of other enzymatic reactions, the developed technology could be the basis for creating a new generation of acousto-electronic biosensors.

LB films are widely used in life science research due to having properties similar to those of biological films, and the sensitivity and selectivity of bionomics sensors can be enhanced by doping other biomolecules or nanomaterials into LB films. Solanki et al. [64] constructed a sensor for the detection of dengue fever by transferring an LB film of molybdenum disulfide (MoS_2_) and gold nanoparticle (Au NP) composites to an electrode coated with ITO electrodes and immobilized an antibody specific for dengue non-structural protein 1 (NS1) antigen on the LB film to construct a dengue detection sensor. 

Atomic force microscopy (AFM) with functionalized tips is a leading technique for measuring local adhesion forces through single-molecule force spectroscopy (SMFS). Marcuello et al. [65] developed a novel approach using the LB technique that was applied for lever functionalization with cellulose nanocrystals and compared it to traditional chemical methods. The LB method demonstrated almost six times greater efficiency in cellulose nanocrystal coverage than chemical techniques. Additionally, LB technology does not require linker molecules, which can otherwise cause an overestimation of interaction forces. The structural characterization and SMFS measurements of lignocellulosic polymers indicate that the LB approach allows for precise control of lever coverage, enhancing the accuracy of adhesion measurements. This methodology is anticipated to significantly influence AFM tip and tipless functionalization and SMFS measurements across various fields. Johnson et al. [66] prepared cellulose sulfate nanofibers (CSNFs) by the sulfonation of virgin jute fibers using chlorosulfonic acid (CSA). It was found that CSNFs could maintain high ammonia adsorption capacity over a wide acidic pH range (2.5 to 6.5).

**Figure 12:**
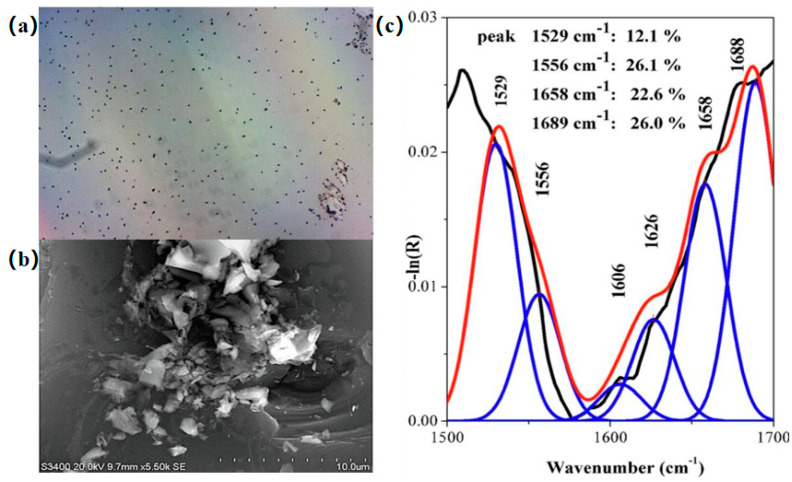
(**a**) Typical optical images at 500× for Aβ(1-42) deposited by LB on Si covered by a C_60_ layer. (**b**) SEM images at low and high magnifications of Aβ(1-42) on Si covered by a C_60_ layer L-B. (**c**) Example of the FTIR reflectance spectra deconvolution for L-B films Aβ(1-42) on Si covered by a C_60_ layer [67]. Copyright 2021, Elsevier.

**Figure 13:**
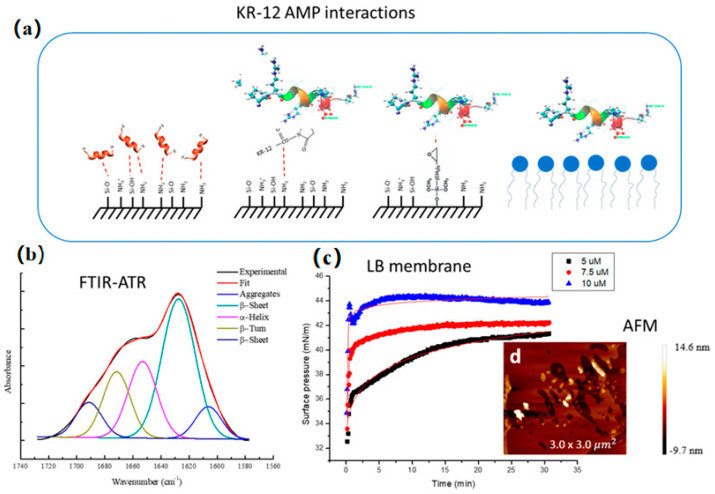
(**a**) The interactions of KR-12 AMP. (**b**) FTIR-ATR results: Deconvolution of the amide I signal for the lyophilized. (**c**) Stability curves of the Langmuir mixture films in the presence of KR-12 peptide at three different concentrations, and (**d**) an AFM image of lower KR-12 MIC [68]. Copyright 2022, Elsevier.

Meanwhile, LB technology also has an important medical prospect in the prevention and treatment of Alzheimer’s disease (AD). Breazu et al. [67] found that the inhibition of the fibrillar denaturation of Aβ could better prevent and treat AD by investigating the effect of Aβ amyloid aggregation based on LB technology (Figure 12). Chanci et al. [68] synthesized KR-12 antimicrobial peptide (AMP) antimicrobial peptide using LB technology and tested its activity to study the antibacterial mechanism against Staphylococcus aureus. The results show lipid film perturbation at lower KR-12 MIC (Figure 13).

LB film technology provides an ideal model for biofilm research, which can study the film-forming properties of organic molecules at the molecular level and obtain the main parameters, such as the distance between organic molecules in the state of ordered arrangement. This is of great significance for the in-depth study of the bioorganic chemical reaction mechanism occurring on the biofilm surface.

## 6. Conclusions

Serving as an advanced interfacial nanomaterial assembly technique, LB technology enables precise control and assembly of customized nanostructures at interfaces on a molecular scale. We provide a succinct overview of the evolution and applications of LB film technology in various fields including gas sensors, electrochemistry, biomimetic films, and interfacial nanostructures. 

The LB technique offers significant advantages in the field of gas sensors, particularly in enhancing sensor performance. This technique allows for highly controllable film structures, enabling the creation of films with precisely ordered molecular arrangements, which is crucial for improving sensitivity and selectivity. It supports the combination and doping of various materials, such as carbon nanomaterials, nanoparticles, conductive polymers, and phospholipids, facilitating the design of multifunctional sensor films. The LB technique can produce ultra-thin films, ranging from a few nanometers to tens of nanometers in thickness, thereby enhancing the response speed and sensitivity of sensors. Additionally, it optimizes the surface properties of the films, improving interactions with target gases and enhancing detection performance. While the LB technique significantly enhances gas sensor performance, addressing its complexity, cost, stability, applicability, and reproducibility challenges is crucial for broader industrial application. LB technology presents significant advantages in the field of electrochemistry. The ordered molecular arrangements enhance electrode surface properties and electrochemical reaction efficiency. LB technology can increase the surface area electrode and the reaction rates. This technology also facilitates the study of interfacial electron transfer dynamics, as it can precisely control the surface state of electrodes. LB technology offers significant advantages in the field of biomimetic membranes. LB films can achieve ultra-thin thicknesses from a few nanometers to tens of nanometers, essential for applications like biosensors and drug delivery systems. LB technology supports the development of advanced biomimetic devices and systems, such as artificial organs and tissue engineering scaffolds, by providing reliable methods to create biocompatible membrane structures. The future of Langmuir–Blodgett technology holds great potential for scientific and technological advances across multiple disciplines, making it a key area of focus for researchers and innovators.

The research trajectory and future prospects of Langmuir–Blodgett (LB) technology are promising. Initial studies focused on fundamental theoretical exploration and method optimization. As the technology advanced, researchers began experimenting with various materials, such as nanoparticles, polymers, and biomolecules, expanding the potential applications of LB films. Recent applications include highly sensitive and selective sensors, biocompatible biomedical devices, and efficient energy storage systems. Efforts to improve the fabrication process have enhanced the stability and consistency of LB films, making them more practical for real-world applications. Future prospects include multifunctional sensors, biomedical applications, energy devices, environmental monitoring, and advanced nanomaterials, highlighting LB technology’s broad and impactful potential.

## Figures and Tables

**Table 1:** Summary of comparisons between Langmuir–Blodgett and Langmuir–Schaefer.

Comparison Criteria	Langmuir–Blodgett (LB) Technique	Langmuir–Schaefer (LS) Technique
Basic Principle	Forms monolayers or multilayers at the air–water interface and transfers them to a solid substrate by vertically lifting or lowering the substrate.	Forms monolayers at the air–water interface and transfers them to a solid substrate by horizontal contact.
Transfer Process	Vertical transfer, in which the substrate moves perpendicular to the air–water interface.	Horizontal transfer, in which the substrate moves parallel to the air–water interface.
Film Structure Control	Allows for precise control over the number of layers and molecular arrangement and is suitable for creating highly ordered multilayer structures.	Primarily used for creating monolayers or a few layers, with less control over molecular arrangement.
Application Fields	Electronic devices, sensors, biomimetic membranes, etc.	Monolayers, surface modification, functionalization, etc.
Advantages	Enables precise control over the film’s thickness and arrangement and is suitable for complex multilayer structures.	Gentle transfer process and less damaging to molecular films and is suitable for softer or unstable molecular films.
Disadvantages	The transfer process can introduce defects, especially for softer or unstable molecular films.	Difficult to precisely control multilayer structure and arrangement and is not suitable for complex multilayer structures.
